# McCune-Albright syndrome and the extraskeletal manifestations of fibrous dysplasia

**DOI:** 10.1186/1750-1172-7-S1-S4

**Published:** 2012-05-24

**Authors:** Michael T Collins, Frederick R Singer, Erica Eugster

**Affiliations:** 1 Skeletal Clinical Studies Unit, Craniofacial and Skeletal Diseases Branch, National Institute of Dental and Craniofacial Research, National Institutes of Health, Bethesda, MD, USA; 2 Director Endocrine and Bone Disease Program, John Wayne Cancer Institute, Santa Monica, CA, USA; 3 Section of Pediatric Endocrinology, Department of Pediatrics, Indiana University School of Medicine, Indianapolis, IN, USA

## Abstract

Fibrous dysplasia (FD) is sometimes accompanied by extraskeletal manifestations that can include any combination of café-au-lait macules, hyperfunctioning endocrinopathies, such as gonadotropin-independent precocious puberty, hyperthyroidism, growth hormone excess, FGF23-mediated renal phosphate wasting, and/or Cushing syndrome, as well as other less common features. The combination of any of these findings, with or without FD, is known as McCune-Albright syndrome (MAS). The broad spectrum of involved tissues and the unpredictable combination of findings owe to the fact that molecular defect is due to dominant activating mutations in the widely expressed signaling protein, G_s_α, and the fact these mutations arises sporadically, often times early in development, prior to gastrulation, and can distribute across many or few tissues.

The complexity can be mastered by a systematic screening of potentially involved tissues and cognizance that the pattern of involved tissues is established, to some degree, in utero. Thorough testing allows the clinician to establish, often times at presentation, the full extent of the disease, and importantly as well what tissues are unaffected. Treatment and follow-up can then be focused on affected systems and a meaningful prognosis can be offered to the patient and family. The authors outline screening and treatment strategies that allow for effective management of the extraskeletal manifestations of FD.

## Introduction

The original extraskeletal manifestations of fibrous dysplasia (FD) reported by McCune [1] and Albright [2] were café-au-lait spots, precocious puberty, and hyperthyroidism. With time a number of manifestations were added to the spectrum of findings that could be seen in association with FD. These included growth hormone (GH) excess [3], hypercortisolism [4], hypophosphatemia/osteomalacia [5], hepatic involvement [6], cardiac involvement [7], and others [8].

## NIH cohort

To evaluate the extraskeletal manifestations observed in patients with FD we reviewed all of the patients seen at the National Institutes of Health over the last 24 years. The evaluation included physical examination, imaging studies (skeletal survey, head CT, nuclear medicine bone scan, ultrasound of the thyroid and gonads, and MRI of the pituitary), biochemical studies of skeletal metabolism and endocrine axes, and when available mutation analysis of affected tissue. There have been 140 patients evaluated at the time of this review. Patients have been followed from <1 – 24 years.

## Prevalence of extraskeletal manifestations

The relative prevalence of findings in MAS patients in the NIH cohort are shown in Table 1. While these data probably reflect the relative prevalence of each of these findings, it is also likely that the NIH cohort represents a more severely affected group of patients than is typically found in clinical practice. Therefore the likelihood of an individual patient with FD having a given manifestation is probably lower than shown here.

**Table 1:** Prevalence of major findings in the NIH cohort of patients with fibrous dysplasia/McCune-Albright syndrome

Clinical finding	% patients^1^
Fibrous dysplasia	98
Café-au-lait spots	66
Gonadal abnormalities	
Male: (ultrasound)^2^	70
Female: precocious puberty	50
Thyroid abnormalities	
Abnormal ultrasound (U/S)	66
Hyperthyroid + abnormal U/S	28
Renal phosphate wasting	43
Hypophosphatemia	10
Growth hormone excess	21
Cushing’s syndrome	4

^1^ n = 140; 58 males, 82 females

^2^ detected on ultrasound

In addition to the major and more common/classic findings seen in association with FD as part of the McCune-Albright syndrome, we have observed a number of other findings in associated with the disease. These are shown in Table 2.

**Table 2:** Prevalence of less common findings in the NIH cohort of patients with fibrous dysplasia

Other clinical findings	% patients^1^
Gastrointestinal	7
History of hepatitis^2^	4
Reflux^2^	5
Pancreatitis^2^	3
Polyps^3^	5
Cardiac	6
Tachycardia^4^	4
Aortic root dilatation (GH excess)^5^	2
Hematologic	1
Platelet dysfunction	1
Cancer	4
Thyroid^6^	1
Breast^6^	2
Bone^6^	1
Testicular^6^	1
Hyperparathyroid	1
Neuropsychiatric	9

^1^n = 140; 58 males, 82 females, ^2^appeared in childhood, common causes excluded, ^3^atypical, upper GI tract polyps, myxomatoid pathology more common, ^4^unexplained/not associated with hyperthyroidism, ^5^only seen in patients with growth hormone excess, ^6^all tumors bear G_s_α mutation, adjacent normal tissue mutation negative.

## Timing of appearance of extraskeletal manifestations

An important consideration in terms of patient/family counseling and the ability to give a prognosis for patients with FD/MAS is when are the manifestation of the disease established, and its corollary question, when is it safe to say that a given aspect of the disease will not manifest. The answer to these questions depends upon early and complete screening to establish if a tissue is affected or not. Whether or not a tissue is affected is also important for long term follow-up. In a study by Hart et al., using the combined tools of ^99^Tc-MDP bone scans, skeletal surveys, and CT scans of the skull, we were able to establish that the majority of skeletal disease, depending on the site, was established roughly between 3 – 10 years of age. Almost all sites of disease that eventually became clinically significant were present by the age of 5 (Table 3). In most cases, this means that almost all clinically significant disease will be present at the time of the first evaluation.

**Table 3:** Age at which FD lesions are established by site

Percent of lesions present	Craniofacial	Axial	Extremity	Total body
50%	NA	7.4	4.9	5.7
75%	NA	11.7	9.6	10.7
90%	3.4	15.5	13.7	15

NA = not applicable

**Figure 1:**
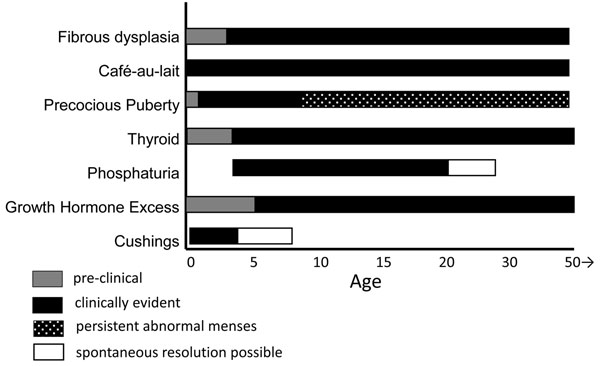
The relative age at which any given aspect of the disease becomes clinically evident is depicted by a solid black bar. Preclinical disease is depicted by gray bars, and ages during which spontaneous resolution is possible for Cushing’s disease and phosphaturia are shown in open bars. The period of time during which abnormal menstruation can be expected is depicted by the stippled bar.

While not as well studied, this same pattern of the early establishment of disease is probably true for the extraskeletal manifestations. For example, by ultrasound, the most sensitive tool for the detection of thyroid disease, involvement of the thyroid, or lack thereof, is established at the first evaluation and persists over many years of follow-up. These findings are consistent with our current understanding of the molecular genetics and embryology of the disease. The manifestations of FD/MAS are due to somatic activating mutations in the *GNAS* gene, sometimes referred to as the *gsp* oncogene, which codes for the protein G_s_α that is involved in intracellular cAMP production [9,10]. To result in a disease that involves cells derived from all three germ layers (ectoderm – e.g. skin, mesoderm – e.g. bone, and endoderm – e.g. thyroid), the mutation must occur very early in development. Thus the “map” of involved tissues, many of which will not become clinically evident for some time, are determined in utero. With the exception of Cushing’s syndrome, phosphaturia, and precocious puberty, in the vast majority of cases once a manifestation is present, it exists throughout life. Figure 1 depicts graphically what one can expect, as far as the age at which clinically significant disease becomes evident.

## Café-au-lait spots

**Figure 2:**
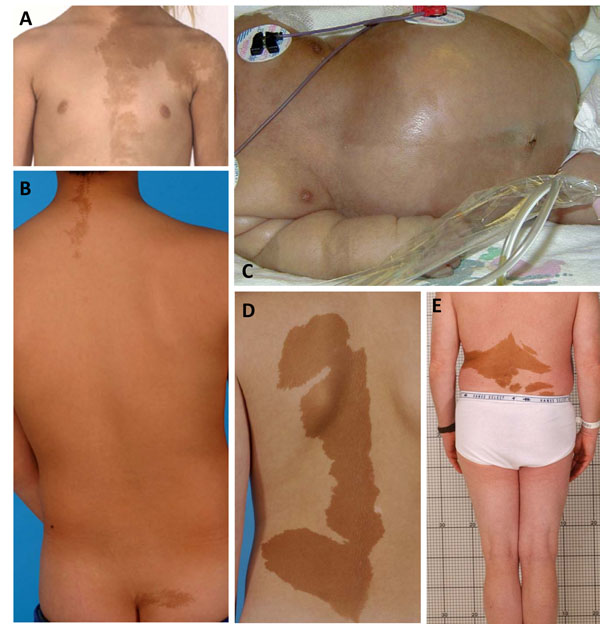
Representative Café-au-lait Spots Seen in McCune-Albright Syndrome. A spectrum of spots is shown; Panels A & B demonstrate “classic” spots that both respect the midline and display “coast of Maine” borders. Panel C shows a very unusual spot seen in a child with MAS and neonatal Cushing syndrome. While the spot respects the midline, the borders are smooth and the spots alternate from left to right in a harlequin pattern. Panel D depicts a very large spot with relatively smooth borders seen in a patient with relatively little FD. Panel E demonstrates a spot that clearly does not respect the midline.

When present, the café-au-lait spots that can be seen in MAS are typically the first manifestation of the disease, usually appearing either at or shortly after birth. As such, they can be an early clue to the diagnosis. They have been classically described as having a “coast of Maine” border, which refers to the jagged appearance of the Maine coastline as it appears on maps. While this is usually the case, it is not always true. Examples of café-au-lait spots seen in MAS that both conform with and defy this dictum are shown in Fig. 2. Likewise, café-au-lait spots found in MAS usually show some association with (“respect”) to the midline. Again, while this is often the case, there are frequent exceptions. Examples of these can be seen in Fig. 2 C & E. While these spots do cross the midline, they retain some association to the midline.

Contrary to what has been previously reported, we have not observed a correlation between the size of the spots and the extent of the disease. Nor have we observed a correlation between side of the body on which the spot is found and the side of the body on which the FD is found.

The café-au-lait spots seen in association with FD are the result of *gsp*-bearing melanocytes in which the mutation brings about c-AMP-mediated tyrosinase gene activation and melanin production in mutation-bearing cells [11]. There are no well-defined effective treatments for the hyperpigmentation seen in MAS. Attempts to bleach areas of hyperpigmentation usually leave an area of under pigmentation, which is usually unsatisfying to the patient. A single report of the efficacy of Q-switched ruby laser in the treatment of the café-au-lait spots of MAS has been reported [12], but further evidence of efficacy is necessary before such a treatment can be routinely recommended.

## Precocious puberty

### Introduction

Precocious puberty is one of the defining manifestations of McCune-Albright syndrome (MAS) (10). It arises due to autonomous *gsp*-mediated gonadal function in cells harboring the *GNAS* activating mutation. Thus, it is characterized as a form of peripheral precocious puberty, in contrast to the early hypothalamic-pituitary-gonadal (HPG) axis activation designated as central precocious puberty. Although it might theoretically be expected to affect girls and boys equally, precocious puberty in children with MAS is far more common in girls, in whom it is typically both the presenting feature as well as the one that ultimately leads to the diagnosis being made. As the clinical characteristics, diagnosis and treatment are distinctly different, precocious puberty in girls will be considered separately from precocious puberty in boys with MAS.

### Precocious puberty in girls

The typical presentation of precocious puberty in girls with MAS consists of vaginal bleeding. Typically painless, sometimes profuse, and usually accompanied by the development of breast tissue; this represents withdrawal bleeding following the resolution of large unilateral estrogen-producing ovarian cysts [11]. Since the cysts are usually asymptomatic, their presence often goes unrecognized until the bleeding occurs. On physical exam, most girls are noted to have mild breast enlargement at a Tanner II or III stage of development. If the child does not come to attention until after the resolution of the initial episode, the breast tissue may have resolved and on casual inspection involuted breast tissue may be missed. Similarly, the finding of obvious and classic café-au-lait macules in girls with MAS is quite variable and, even if present, their significance may go unrecognized. Therefore, it is not unusual for girls to present initially to an emergency department or primary care clinic and have the treating physicians fail to include MAS in the differential diagnosis. As some of the clinical and radiographic findings overlap with those of juvenile granulosa cell tumors, girls with MAS sometimes end up undergoing unnecessary oophorectomy for a presumed ovarian tumor [12]. Ideally, vaginal bleeding in a prepubertal girl should always prompt consultation with a pediatric endocrinologist so that, in the case of MAS, unneeded loss of the ovary can be prevented.

In addition to a history and physical exam, the initial evaluation of precocious puberty in a girl with suspected MAS consists of laboratory and radiographic studies. Classic biochemical findings include elevated estradiol and estrone levels, which are many-fold higher than prepubertal values, in association with suppressed gonadotropins. Pelvic ultrasound typically reveals a large unilateral ovarian cyst which may be hemorrhagic and appear to have mixed cystic and solid elements. As would be predicted, extreme asymmetry in ovarian volumes between the two sides is the norm, in striking contrast to the symmetrical ovarian enlargement emblematic of central precocious puberty [13]. If seen after the initial episode, growth parameters and bone age x-ray are often normal. The diagnosis of MAS is typically made clinically on the basis of classic features, including café-au-lait pigmentation. A bone scan to look for fibrous dysplasia and screening for other MAS-associated endocrinopathies are important elements of the diagnostic work-up. However, isolated precocious puberty without any other identifiable abnormalities may also be seen in girls with MAS [14]. Serial ultrasounds, if indicated, will reveal a gradual resolution of the ovarian cyst over several weeks.

The natural history of precocious puberty in girls with MAS is extremely variable. The first episode can occur as early as during the first few months of life or as late as age 6 or 7 years. Likewise, subsequent episodes are highly unpredictable. While many girls have extended periods of quiescence that last for several years, others have frequent bouts of vaginal bleeding along with progressive breast development followed by the onset of pubic and axillary hair and adult body odor. As is seen with all forms of significant sex steroid exposure during the prepubertal years, linear growth acceleration and advanced skeletal maturation also ensue. Unfortunately, there is no way to predict exactly when the next episode of precocious puberty will occur, which can contribute to the anxiety experienced by parents when this complex disorder is diagnosed. Similarly, the precocious puberty flare-ups themselves vary in severity. In contrast to the typical vaginal bleeding, some girls are noted to simply have periodic waxing and waning of breast enlargement without overt bleeding.

Historically, the prevailing notion was that the HPG axis would override autonomous ovarian function in girls with MAS once physiologic puberty was underway. However, this has given way to the recognition that women with MAS continue to experience intermittent autonomous ovarian function marked by the development of large unilateral ovarian cysts and irregular vaginal bleeding [15]. This has the potential to interfere with normal ovulatory function with subsequent implications in terms of fertility [16]. However, in most cases adults with MAS have been able to have children, even if it may take longer than normal to conceive.

### Management

Clinical management of precocious puberty in a girl with MAS consists initially of observation. Girls with only sporadic and infrequent vaginal bleeding often do not need to be treated. In the subset of girls with a progressive form of precocious puberty, pharmacologic intervention is recommended in order to prevent early epiphyseal fusion and augment adult height. However, other than anecdotal case reports, to what extent height is compromised and whether intervention ameliorates this, is not well established. As is the case for all aspects of MAS, both the rarity and heterogeneity of the disease present significant challenges to rigorous investigation.

Current treatment of precocious puberty in girls with MAS revolves around the use of anti-estrogens. Two basic strategies exist. The first relies on interfering with estrogen biosynthesis through the use of an aromatase inhibitor [17], while the second aims to blunt the effects of estrogen at the level of the end-organ through receptor blockade. To date, small uncontrolled trials have been conducted with first, second and third generation aromatase inhibitors. Experience with the first generation agent, testolactone, was ultimately marred by sub-optimal efficacy as well as issues with compliance [18]. Investigation of the second generation aromatase inhibitor, fadrozole, was abandoned following concerns about adrenal suppression [19]. Among the third generation compounds, anastrozole has been deemed ineffective [20]. Letrozole, however, was found to result in a significant decrease in rates of skeletal maturation in a small number of girls treated for 3 years, although mean ovarian volumes were unchanged [21]. Most girls also experienced a decrease or cessation in vaginal bleeding while on letrozole, although one subject who had entered secondary central precocious puberty developed a large cyst with subsequent ovarian torsion. Treatment with the selective estrogen receptor modulator, tamoxifen, has also been studied in a group of girls with MAS treated for one year. In addition to a significant decrease in vaginal bleeding, tamoxifen resulted in an improvement in growth velocity and bone age advancement [22]. Despite these positive results, the finding of increased uterine and ovarian volumes in the girls treated with tamoxifen represents a potential safety concern that to date remains unresolved. Lastly, preliminary results from a prospective study utilizing the pure estrogen receptor blocker, fulvestrant, are available. A decrease in the median number of vaginal bleeding days as well as in the average rate of skeletal advancement in 30 girls treated for one year was seen [23]. Thus, relatively comparable efficacy has now been observed with several agents used in the treatment of precocious puberty in girls with MAS, although none have been perfect and none have emerged as being clearly superior to the others. Studies comparing available medications in a head to head fashion are needed.

### Precocious puberty in boys

There are several important differences between precocious puberty in girls with MAS and its counterpart in boys. One distinction is that precocious puberty is very rare in affected boys, who are diagnosed with MAS far more often due to the finding of bone disease or café-au-lait pigmentation. An additional dissimilarity is that the precocious puberty, when present, is more likely to be subtle and indolent in boys. Lastly, the activating G_s_α mutation and resulting gonadal hyperfunction have been reported to be limited to the testicular Sertoli cells in several boys with MAS. This has resulted in either unilateral or bilateral macroorchidism without precocious puberty [24][25][26][27]. Interestingly, many of these cases have also been associated with testicular microlithiasis, which has also been identified in males of all ages with MAS [28][29]. Due to its extreme rarity, only anecdotal case reports detailing treatment options for precocious puberty in boys are available. The most common approach employs combination therapy in the form of an androgen receptor blocker such as spironolactone, flutamide or cyproterone acetate along with a compound that interferes with sex steroid synthesis such as ketoconazole or an aromatase inhibitor [30]. On principle, the same strategies used to treat boys with other forms of peripheral precocious puberty such as familial male precocious puberty, would be efficacious in the setting of MAS. One such example is the combination of bicalutamide, a pure androgen receptor blocker, with the third generation aromatase inhibitor anastrozole [31]. Similar to what has been reported in women with MAS, fifteen year follow-up in a boy with MAS and history of precocious puberty indicated persistent autonomous testicular hyperfunction and suppressed gonadotropins [32]. Although inhibin B was undetectable, active spermatogenesis occurred and was seemingly unaffected.

## Thyroid

At the NIH approximately 2/3 of the patients had involvement of the thyroid when assessed by the most sensitive method for assessing thyroid involvement, ultrasound [13]. Only about 1/2 of the patients who had involvement of the thyroid detected on ultrasound had frank hyperthyroidism, as evidenced by a suppressed TSH. As in every aspect of MAS, the thyroid findings exist along a spectrum from an isolated area seen on ultrasound with no clinical findings to patients with obvious goiters, and hyperthyroidism that is unable to be adequately controlled with medications and requires either surgery or ablation. The presence of the *gsp* mutation in thyroid tissue results in ligand-independent activation of the TSH/G-protein/cAMP pathway, which is known to result in both hyperplasia and hyperfunction [14]. Additionally, the *gsp* mutation results in increased thyroxine (T4) to triiodothyronine (T3) conversion, which accounts for the T3-dominant biochemical phenotype of MAS patients with hyperthyroidism [13].

**Figure 3:**
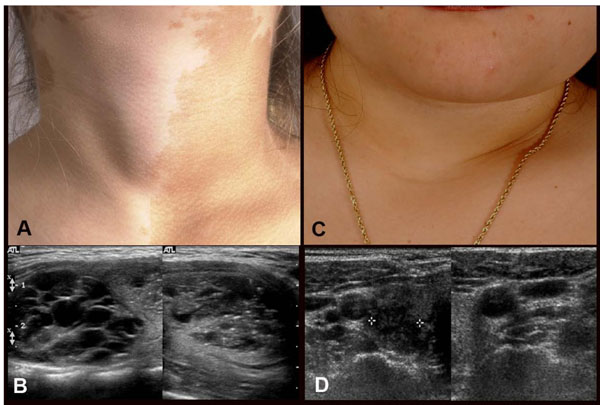
Clinical and ultrasound findings of thyroid involvement in FD/MAS. Panels A & B demonstrate the findings in a 9-year-old girl with MAS and hyperthyroidism. A goiter is clearly seen on inspection (A) and the ultrasound (B) shows the typical cystic (Swiss cheese) appearance that seen in MAS thyroids. Panels C & D demonstrate the findings of a 30-year-old woman with MAS. While no goiter was evident on inspection, nor was one obvious on palpation, the ultrasound clearly demonstrated the typical findings seen in ultrasounds of patients with MAS and thyroid involvement. Adapted from reference [13.]

It is important to diagnose hyperthyroidism in MAS, as hyperthyroidism can advance bone age, which may already be a problem in children with precocious puberty, lead to or exacerbate osteoporosis, and cause a plethora of other metabolic derangements. Diagnosis is usually straight forward and involves the measurement of TSH and thyroid hormones, T3 and T4. It is not uncommon to have a normal T4 in the setting of a suppressed TSH. This apparently incongruous finding is clarified when T3 is measured and found to be high. In patients in whom the only abnormality is an abnormal ultrasound, it is important to continue to check TSH and thyroid hormone periodically as the development of frank hyperthyroidism may occur later. The ultrasound findings in MAS are usually a mixture of mostly cystic with some solid lesions (Fig. 3) [13,15].

Hyperthyroidism in MAS usually responds quite well to thionamides. However, since hyperthyroidism is one of the aspects of MAS that persists, it is often desirable for the patient to undergo definitive treatment, which usually means surgery or ablation with radioactive iodine. Surgery may be difficult in very small children, and is therefore recommended to delay surgery in small children.

## Hypophosphatemia

**Figure 4:**
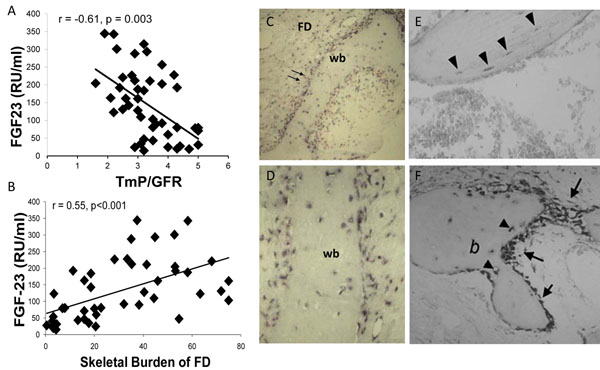
Panel A demonstrates the relationship and significant negative correlation between serum FGF23 and the degree of phosphate wasting, supporting the concept that FGF23 is responsible for the phosphate wasting that can be seen in association with FD. Panel B depicts the relationship and significant positive correlation between the serum FGF23 and the skeletal burden of FD, which supports the hypothesis that the FD tissue is the source of FGF23. Panels C&D are low and high power views, respectively, of in situ hybridization studies using probes for FGF23 that demonstrate that FD tissue demonstrates high levels of FGF23 transcripts. Panels E&F show high levels of FGF23 transcripts in bone from control, normal bone; E osteocytes and F osteoblasts in healing fracture callus. FD = fibrous dysplasia, wb = woven bone, b = bone, double arrows designate FD stromal cells, arrow heads designate osteocytes, single arrows designate osteoblasts. Adapted from reference [17].

While rickets in association with FD was originally reported in 1968 [5], it was not until 2001 that it was evident the cause was a circulating phosphaturic hormone, similar to what is seen in the inherited forms of rickets [16]. Overproduction of FGF23 by FD tissue was found to be the cause [17]. FGF23 is overproduced by FD tissue, such that the greater the disease burden, the higher the FGF23, the greater the degree of renal phosphate wasting, and the lower the serum phosphorus (Fig. 4). Therefore, significant hypophosphatemia is only seen in patients with a very significant skeletal burden of FD. It has also been observed that, unlike many other extraskeletal manifestations aspects, renal phosphate wasting can spontaneously resolve as patients age. This probably reflects intrinsic changes that have been observed at the tissue level and characterized as “normalization” [18].

The clinical sequelae and significance of hypophosphatemia are an earlier age of first fracture, more fractures, and bone pain [19]. There are no controlled studies to support that treating hypophosphatemia decreases fractures or improves pain, but observation of treated patients suggests that treatment may improve outcomes. Treatment of hypophosphatemia is the same as in other FGF23-mediated phosphate wasting disorders, and involves the use of phosphate and active vitamin D (calcitriol or alfacalcidiol). Details for this treatment regimen can be found elsewhere [8,20].

## Growth hormone excess

Growth hormone excess in association with FD is the manifestation of *gsp* mutation in the anterior pituitary [21]. It is always accompanied by skull base FD, and the vast majority of patients also have hyperprolactinemia. The usual presenting sign is increased growth velocity. However, if GH excess is accompanied by precocious puberty, the clinical sign of increased growth velocity can be obscured by the increase in growth velocity that is seen as part of precocious puberty. Likewise, if a patient with precocious puberty achieves his/her predicted height, this can be a sign of GH excess, as precocious puberty should have resulted in short stature. For this reason, it is important to do laboratory screening for GH excess in patients with FD, as the clinical evaluation can be confounded by concomitant hormonal excess and make the clinical exam difficult to interpret.

Probably the most important reason to diagnose GH excess in association with FD is that it is associated with an increase in morbidity, specifically in the craniofacial region. GH excess in FD is associated with macrocephaly and vision loss [22,23].

The diagnosis of GH excess is usually straightforward. Non-suppressible serum GH on an oral glucose tolerance test (OGTT) is diagnostic of GH excess [21]. However, in subtle disease the results of the OGTT can be equivocal, especially in young children [24]. In these difficult cases, frequent (every 20 min) overnight sampling may be of utility. Children with GH excess will fail to have any intervals when the GH value is below 1 ng/ml. As stated previously, almost all patients with FD/MAS- associated GH excess also have an elevate prolactin. Therefore, the prolactin level can be an additional tool in either confirming or excluding the diagnosis of GH excess.

Our most recent analysis of the NIH cohort of patients with GH excess indicates that early diagnosis and treatment of GH excess may prevent GH excess-associated morbidity, specifically vision loss.

Treatment of GH excess in FD/MAS is almost exclusively confined to medical treatment. Usually, due to the massive expansion of the skull base with FD, which includes obliteration of the sphenoid sinus, the traditional transphenoidal approach to the pituitary is either not possible or extremely difficult. An additional important consideration if surgery is contemplated, is the fact that, in spite of what may appear as a single adenoma on pituitary imaging, the entire anterior pituitary is usually infiltrated with areas of herplastic and/or adenomatous somatotrophs and somatolactotrophs. The implications of this finding are that surgical cure of GH excess in FD/MAS will require a complete hypophysectomy – a treatment that, if it is to be embarked upon, is probably best delayed until young adulthood. Given that treatment doses of radiation directed at FD are associated with an increase in malignant transformation [25,26], radiation is rarely an acceptable approach.

### Treatment

The drug with which there is the longest experience in treating FD/MAS-related GH excess is octreotide [21,27-29]. It is usually effective in lowering serum GH and IGF-1 levels. In growing children, the goal of treatment is to decrease the IGF-1 to the middle of the normal range (IGF-1 Z-score = 0). In mature patients, the goal is to decrease the serum IGF-1 to as low as possible. The GH receptor antagonist, pegvisomant, has also been shown to be effective in treating MAS patients with GH excess [30,31]. Which drug is superior is not known. In some patients a combination of both octreotide and pegvisomant is necessary to achieve control, and in a small minority of patients not even the combination is effective. This is the group that should be considered for surgery and/or radiation. We have attempted to treat the GH excess in MAS with the dopamine agonist, cabergoline as a single agent in several cases, but had no success (unpublished data).

The hyperprolactinemia that usually accompanies GH excess in MAS is not affected by treatment with octreotide or pegvisomant, but is almost always effectively controlled with dopamine agonists, such as cabergoline or bromocriptine.

## Cushing’s syndrome

Cushing’s syndrome is the rarest of endocrine abnormalities found in MAS [32]. It always occurs in the neonatal period, which parallels the involution of the fetal adrenal gland and may suggest a differential effect of the *gsp* mutation on the fetal adrenal, which is supported by the fact that both glands are always involved [33]. Cushing’s syndrome is one of the few aspects of MAS that is associated with increased and early mortality. Most of the early mortality associated with Cushing’s syndrome in MAS is due to opportunistic infections, and highlights the importance of prophylactic treatment, notably for *Pneumocystis species*. Cushing’s syndrome usually only occurs in patients with MAS with significant involvement of multiple other tissues. Patients with Cushing’s are also more likely to have many of the manifestations mentioned in Table 2.

A review of all the published cases of Cushing’s syndrome in MAS [32] listed the following signs and symptoms: small for gestational age (50%), round facies (67%), failure to thrive (60%), hypertension (33%), nephrocalcinosis (30%), hirsutism (27%), hyperglycemia (20%), and linear growth arrest (10%). While it is clearly documented that some cases of Cushing’s syndrome can resolve spontaneously [34], it is impossible to predict in which patients this will occur. Therefore making the diagnosis necessitates treatment. This usually involves surgical removal of diseased adrenal glands. However, medical treatment is sometimes able to lower serum cortisol to normal or lower. Since many children with MAS and Cushing’s syndrome also have evidence of a cholestatic hepatitis, the often effective drug ketoconazole is avoided due to it is potentially hepatotoxicity. Metyrapone is frequently effective. The initial dose is 300 mg/m2/day. It may be increased to as high as 1200 mg/m2/day, as needed. In particularly sick children, medical treatment with metyrapone may buy time until the child is healthy enough for surgery.

Long term sequelae of Cushing’s syndrome in MAS include a significantly increased prevalence of cognitive disorders, including specific learning or speech disorders, or global developmental delay and speech apraxia.

## Other extraskeletal manifestations

The additional less common extraskeletal manifestations associated with MAS are outlined in Table 2. Some will be discussed below. Hepatitis, when it occurs, is more pronounced after birth, has laboratory manifestations consistent with cholestasis, progressively wanes with age, usually persists into adulthood, albeit mild, and is virtually never associated with a functional defect in synthesis of important hepatic factors [6].

### Gastrointestinal reflux

Gastroesophageal reflux is infrequently seen in MAS, and primarily in patients with multiple extraskeletal manifestations. It usually manifests in childhood and can be a source of significant discomfort to patients. The etiology is unknown, but is presumed to be incompetence of the lower esophageal sphincter from unknown mechanisms. Hyperacidity does not appear to be the primary issue. Treatment is usually medical and involves the use of histamine blockers or proton pump inhibitors. It has not been reported to be associated with metaplasia of the lower esophagus (Barrett’s esophagus).

### Gastrointestinal polyps

Gastrointestinal polyps, especially in unusual locations (gastric and duodenal) and of significant size have been observed in association with MAS (personal observations, MTC). They can become clinically significant if they reach a size that can cause obstruction. The long term significance and malignant potential is unknown, although reports of a role of activating mutations of G_s_α have been seen in association with gastrointestinal malignancies [35]. To date, no gastrointestinal malignancies have been reported in association with FD/MAS.

### Pancreatitis

Idiopathic pancreatitis has been observed in patients with FD/MAS. The prevalence observed in the NIH (approximately 3%) is greater than would be expected in an unselected population, however a direct association with MAS has not been demonstrated and there are no known associations between *gsp* mutations and a predisposition to pancreatitis.

### Cardiac

There are several cardiac abnormalities that have been reported in association with FD/MAS. These include sudden death, tachycardia, high output heart failure and aortic root dilatation. While much has been made of sudden death as part of MAS [7,36], and the fact that the *gsp* mutation was found in cardiac tissue of children who had sudden death, evidence that the cause of death was cardiac, and/or that the *gsp* mutation played a role is lacking. While patients with FD/MAS are clearly at cardiac risk due to hyperthyroidism and other endocrine abnormalities, the risk for sudden cardiac death is probably minimal if any.

### Tachycardia

Tachycardia can be seen in patients with FD/MAS who are hyperthyroid [13,15]. Tachycardia in the absence of hyperthyroidism (unexplained tachycardia) was seen in approximately 4% of the NIH cohort of patients with FD/MAS. There are at least two possible explanations for this; it could represent the presence of the *gsp* mutation in the heart, or it could represent the physiologic response to increased demand placed on the heart due to extensive FD, which is a very vascular tissue. The two explanations are not mutually exclusive. In fact, more extensive extraskeletal involvement is usually seen when there is extensive skeletal involvement. Therefore, cardiac *gsp*, which has been demonstrated [7] may be more likely to be seen in patients with extensive disease. All of the patients with unexplained tachycardia in the NIH cohort had extensive FD. Therefore, it is not clear if the cause was primary cardiac (*gsp* mutation in the heart), or a secondary, physiologic response to increased demand placed on the heart by extensive bone disease. How to treat these patients is a conundrum. Untreated pathologic tachycardia, as can be seen in hyperthyroidism, can lead to a cardiomyopathy and heart failure. However, inappropriate suppression of physiologically-induced tachycardia that can be seen as part of increased demand could also lead to heart failure. In one patient with total skeletal involvement with FD an effort to suppress demand (vascularity) by aggressive bisphosphonate treatment had no effect – at least in part because it did not appear to have any effect on vascularity. To date, we have opted not to treat these patients with beta blockers. However, they are monitored closely with cardiac echocardiogram and cardiac MRI for any early signs of decompensation suggestive of impending heart failure. Any sign of decompensation will ben and indication for treatment. Thus far, with a follow-up of almost 10 years there has not been any decompensation.

### Aortic root dilatation

We have observed dilatation of the aortic root in several patients with GH excess in the NIH cohort. We have made the assumption that this is the direct effect of GH excess on the heart, as this has been reported in association with acromegaly [37]. In one of the patients the aortic root dilatation is clinically significant. In this subject, who had many years of untreated GH excess and extensive morbidity due to untreated GH excess, the disease led to aortic root dilatation, marked aortic valve insufficiency, atrial dilatation and atrial fibrillation [54]. In the other subjects who started treatment at an earlier age, there has not been any progression and there is no associated cardiac morbidity.

### Platelet dysfunction

Platelet dysfunction has also been reported in association with FD and it has been suggested this may play a role in the extensive bleeding that can be seen during operations on FD tissue [38]. However, FD tissue is also extremely vascular and it is difficult to determine whether platelet dysfunction may contribute to bleeding beyond what is expected from vascularity. Whether or not all patients should be screened for platelet dysfunction is not clear. However, in subjects with a history of difficult to control bleeding, platelet dysfunction should be considered preoperatively.

### Cancer

The cancers that have been reported in association with FD/MAS, and in which the presumably etiologic *gsp* mutation has been identified in the malignant tissue, include malignant transformation of FD, thyroid, and breast. In addition to these, we have received personal communications of cancers of the testes and lung; however these have not been checked for *gsp* mutation. The activating mutations that cause FD/MAS were given the designation as an oncogene (*gsp*) because they were originally found as the cause of benign endocrine adenomas [39]. However these diseases are almost invariably benign and suggest that for malignant transformation to take place additional mutations probably arise in addition to *gsp* mutations. This concept is supported by the finding that *gsp* mutations are not uncommonly seen as part of the genomic landscape of common cancers such as breast and colon, mutations in many other genes known to be associated with cancer development are found [35]. This concept is further supported by the detailed chromosomal and genetic analysis of a cell line that was derived from a patient with FD in whom the FD transformed into a malignant fibrous histiocytoma [40]. In addition to the expected *gsp* mutation there were multiple structural and numerical abnormalities of chromosomes with a large number of unidentifiable chromosomes as well as a p53 mutation in exon 7 accompanied by loss of heterozygosity in the counterpart allele.

### Bone cancers

There are a number of very good reviews that catalogue the reports of cases of FD that have transformed to various types of bone cell-related cancers including, among others osteosarcomas, fibrosarcoma, chondrosarcoma, and even a malignant mesenchymoma that demonstrated multiple cell types all with malignant features [25,26,41-45].

It is difficult to determine what the risk of malignant transformation in FD is from the published literature. Series and centers report the number of cases of cancers, but it is difficult to know what the appropriate denominator is to determine the prevalence, and/or it is difficult to judge what the impact of the referral bias is for that given institution and that series. For example, a review of the Mayo Clinic data identified 28 cases of malignancy out of 1122 total cases, for a prevalence of about 2% [46]. This would be considered by most experts to be a high estimate of the risk of malignant transformation of FD and probably reflects a referral bias of the institution for bone cancers. In the NIH cohort of approximately 140 patients with disease on the more severe end of the spectrum, we have seen only one case in over 20 years of experience, for a prevalence of <1%.

Malignant transformation is suggested by an expanding, previously stable lesion, new focal pain, with the radiographic hallmark being a breach of the bone cortex with the extension of a soft tissue mass beyond the cortex.

In terms of other factors that may impart additional risk (or protection), there is little guidance in the literature. Here the problem is that it is not clear to what extent any individual patient has been studied to identify additional risk factors. It is possible that the presence of GH excess may add additional risk for malignant transformation. The two cases of breast cancer and the single case of malignant transformation of FD observed in the NIH cohort all occurred in women with GH excess. In addition, while not systematically studied, there is a sense from the literature in the patients who appear to have been thoroughly investigated, that GH excess may impart additional risk for the malignant transformation of craniofacial FD [47-50], as well as for bone cancer in general [51,52].

### Thyroid

Thyroid cancer has been observed in two patients in the NIH cohort (prevalence approximately 1.3%). Support for the fact that this was a true relationship between the presence of the *gsp* mutation and thyroid cancer was the fact that in both cases the mutation was found in the neoplastic tissue, but not in the adjacent normal tissue [53]. Further support is lent by the fact that in both cases there were unusual features further suggesting an association, specifically young age and tumor type (clear cell carcinoma, which is a rare variant of thyroid cancer that has been reported in association with hypothyroidism-associated goiter, in which case there will be increased TSH/G_s_α/cAMP signaling).

Diagnosis of cancer within the thyroid of a patient with MAS is difficult, given that the gland is often diffusely abnormal and it is difficult to identify malignant changes on this diffusely abnormal background (Fig. 3). Suggestive clinical findings are an expanding firm nodule, and/or an expanding solid nodule on ultrasound. If these findings are present, a fine needle aspiration should be performed with cytological examination to exclude malignant findings. Given that definitive treatment of hyperthyroidism, which includes thyroidectomy, is often recommended, one should have a low threshold to perform a thyroidectomy on a fine needle aspiration specimen that is inconclusive.

### Breast cancer

Two cases of breast cancer have been reported in association with FD/MAS [54,55]. In neither case did the investigators examine the malignant tissue for *gsp* mutations, so it is not possible to determine whether or not the development of cancer in these women was directly related to the underlying gene defect. In both cases, the women had had precocious puberty, and since prolonged estrogen exposure is known to be a risk factor for the development of breast cancer, it is reasonable to assume that precocious puberty as part of MAS can be considered a risk factor for breast cancer. We have seen two cases of breast cancer in the NIH cohort; both women presented before the age of 30 and both women had had precocious puberty and GH excess. (One of these patients was the patient reported by Huston et al.,[55]) While it is enticing to consider GH excess as an additional risk factor for the development of breast cancer in MAS, it is impossible to say at this point. In fact, whether or not there is a relationship between breast cancer and sporadic GH excess is not clear [56].

### Testicular cancer

While there are no reported cases of testicular cancer in men with MAS, one of the authors has encountered one case (FRS).

### Hyperparathyroidism

While there have been a number of reports of FD/MAS in association with hyperparathyroidism [57-61], in none of these cases was there molecular confirmation, and in the one report where there was a very thorough effort to show molecular confirmation, there was none [62]. This led the authors to conclude that the association of primary hyperparathyroidism with FD/MAS was chance and that hyperparathyroidism did not represent a molecularly-driven association. Furthermore, in reconsidering some of the cases in light of new information, there is a question as to whether the disease described was FD or hyperparathyroidism jaw syndrome (HPT-JT). In HPT-JT, the osseous lesion is a fibroosseous lesion with significant histopathological similarities to FD, and confusion with FD is not difficult. In addition, our current understanding of the molecular regulation of parathyroid function and parathyroid neoplasms does not predict that an activating mutation in G_s_α would lead to hyperparathyroidism or a parathyroid adenoma. For these reasons, most investigators today conclude that hyperparathyroidism should not be considered to be part of the spectrum of FD/MAS.

### Neuropsychiatric

While there has been passing, ill-defined mention of “mental retardation” in association with FD/MAS [63], the most thorough chronicle of a possible association between FD/MAS and any neuropsychiatric problems was the evaluation of the NIH cohort by Brown et al [32]. In this study a number of findings were seen including learning and speech disorders, such as speech apraxia, and global developmental delay. While these findings were seen in approximately 9% of the cohort as a whole, they were found in 44% of the subjects who had had Cushing’s syndrome, indicating that Cushing’s syndrome is a significant risk factor for neuropsychiatric findings in patients with FD/MAS. As Cushing’s syndrome is invariably found in the neonatal period in MAS, it suggests that in utero exposure to high levels of cortisol may be deleterious to brain development. That said it is also possible that Cushing’s syndrome in MAS may be a marker for widespread distribution of the *gsp* mutation and the presence of neuropsychiatric symptoms is a manifestation of central nervous system involvement. In several papers from the Abel laboratory in which the Q227L activating mutation of G_s_α (Q227L), a mutation that is also an activating mutation functionally similar to the R201C/H mutations that cause FD/MAS, was targeted to the central nervous system of mice, the animals developed a spectrum of neuropsychiatric findings including learning disorders [64-70]. One of the more striking findings seen in these mice was the counterintuitive finding that treatment with phosphodiesterase inhibitors seemed to reverse the phenotype. Phosphodiesterases breakdown cAMP, and given that the evidence thus far that much of the pathophysiology of FD/MAS is the direct effect of excess cAMP, one would assume that inhibition of cAMP breakdown would exacerbate, not treat, symptoms of *gsp* expression. Clearly there is much more to learn.

## Summary

From this review, it is clear that the spectrum of extraskeletal manifestations that can be found in MAS is broad – as broad as the tissue distribution of G_s_α expression. While clinicians should consider that almost any finding seen in association with FD/MAS may be the result of tissue-specific *gsp* expression, the majority of the extraskeletal manifestations of MAS are confined to those listed in Table 1. While effective treatments for FD remain elusive, most of the conditions listed in Table 1 are readily amenable to treatment. Given that many of these conditions will worsen the FD if untreated, it is important to suspect, screen for, and treat these extraskeletal manifestations.

This information in this review was presented as part of the Proceedings of the International Meeting on Fibrous Dysplasia/McCune-Albright Syndrome and Cherubism at the National Institutes of Health in Bethesda, Maryland October 3-5, 2010. MTC, FRS and EE have drafted the manuscript. All authors were involved in the critical review of the manuscript. All authors read and approved the final manuscript.

## Competing interests

The authors declare that they have no competing interests.

## References

[B1] McCune DJ Osteitis fibrosa cystica; the case of a nine year old girl who also exhibits precocious puberty, multiple pigmentation of the skin and hyperthyroidism Am J Dis Child 1936 52 743 744

[B2] Albright F Butler AM Hampton AO Smith PH Syndrome characterized by osteitis fibrosa disseminata, areas of pigmentation and endocrine dysfunction, with precocious puberty in females, report of five cases N Engl J Med 1937 216 727 746 10.1056/NEJM193704292161701

[B3] Cremonini N Graziano E Chiarini V Sforza A Zampa GA Atypical McCune-Albright syndrome associated with growth hormone-prolactin pituitary adenoma: natural history, long-term follow-up, and SMS 201-995--Bromocriptine combined treatment results J Clin Endocrinol Metab 1992 75 1166 1169 10.1210/jc.75.4.1166 1400888

[B4] Benjamin DR McRoberts JW Polyostotic fibrous dysplasia associated with Cushing syndrome Arch Pathol 1973 96 175 178 4722878

[B5] Ryan WG Nibbe AF Schwartz TB Ray RD Fibrous dysplasia of bone with vitamin D resistant rickets: a case study Metabolism 1968 17 988 998 10.1016/0026-0495(68)90004-8 5724172

[B6] Silva ES Lumbroso S Medina M Gillerot Y Sultan C Sokal EM Demonstration of McCune-Albright mutations in the liver of children with high gammaGT progressive cholestasis J Hepatol 2000 32 154 158 10673080 10.1016/s0168-8278(00)80202-0

[B7] Weinstein LS Shenker A Gejman PV Merino MJ Friedman E Spiegel AM Activating mutations of the stimulatory G protein in the McCune-Albright syndrome N Engl J Med 1991 325 1688 1695 10.1056/NEJM199112123252403 1944469

[B8] Dumitrescu CE Collins MT McCune-Albright syndrome Orphanet J Rare Dis 2008 3 12 10.1186/1750-1172-3-12 18489744 PMC2459161

[B9] Weinstein LS Shenker A Gejman PV Merino MJ Friedman E Spiegel AM Activating mutations of the stimulatory G protein in the McCune-Albright syndrome N Engl J Med 1991 325 1688 1695 10.1056/NEJM199112123252403 1944469

[B10] Schwindinger WF Francomano CA Levine MA Identification of a mutation in the gene encoding the a subunit of the stimulatory G protein of adenylyl cyclase in McCune-Albright syndrome Proc Natl Acad Sci USA 1992 89 5152 5156 10.1073/pnas.89.11.5152 1594625 PMC49247

[B11] Kim I Kim ER Nam HJ Chin MO Moon YH Oh MR Yeo UC Song SM Kim JS Uhm MR Beck NS Jin DK Activating mutation of Gsa in McCune-Albright syndrome causes skin pigmentation by tyrosinase gene activation on affected melanocytes Horm Res 1999 52 235 240 10.1159/000023467 10844413

[B12] Ozawa T Tateishi C Shirakawa M Murakami E Ishii M Harada T Long-term follow-up of a case of cheek hyperpigmentation associated with McCune-Albright syndrome treated with Q-switched ruby laser Dermatol Surg 2011 37 263 266 10.1111/j.1524-4725.2010.01864.x 21272121

[B13] Celi FS Coppotelli G Chidakel A Kelly M Brillante BA Shawker T Cherman N Feuillan PP Collins MT The role of type-1 and type-2 5'deiodinase in the pathophysiology of the T3 toxicosis of McCune-Albright syndrome J Clin Endocrinol Metab 2008 93 2383 2389 10.1210/jc.2007-2237 18349068 PMC2435649

[B14] Combest WL Russell DH Alteration in cyclic AMP-dependent protein kinases and polyamine biosynthetic enzymes during hypertrophy and hyperplasia of the thyroid in the rat Mol Pharmacol 1983 23 641 647 6306431

[B15] Feuillan PP Shawker T Rose SR Jones J Jeevanram RK Nisula BC Thyroid abnormalities in the McCune-Albright syndrome: ultrasonography and hormone studies J Clin Endocrinol Metab 1990 71 1596 1601 10.1210/jcem-71-6-1596 2229316

[B16] Collins MT Chebli C Jones J Kushner H Consugar M Rinaldo P Wientroub S Bianco P Robey PG Renal phosphate wasting in fibrous dysplasia of bone is part of a generalized renal tubular dysfunction similar to that seen in tumor-induced osteomalacia J Bone Miner Res 2001 16 806 813 10.1359/jbmr.2001.16.5.806 11341325

[B17] Riminucci M Collins MT Fedarko NS Cherman N Corsi A White KE Waguespack S Gupta A Hannon T Econs MJ Bianco P Gehron Robey P FGF-23 in fibrous dysplasia of bone and its relationship to renal phosphate wasting J Clin Invest 2003 112 683 692 12952917 10.1172/JCI18399 PMC182207

[B18] Kuznetsov SA Cherman N Riminucci M Collins MT Robey PG Bianco P Age-dependent demise of GNAS-mutated skeletal stem cells and "normalization" of fibrous dysplasia of bone J Bone Miner Res 2008 23 1731 1740 10.1359/jbmr.080609 18597624 PMC2585500

[B19] Leet AI Chebli C Kushner H Chen CC Kelly MH Brillante BA Robey PG Bianco P Wientroub S Collins MT Fracture incidence in polyostotic fibrous dysplasia and the McCune-Albright syndrome J Bone Miner Res 2004 19 571 577 15005844 10.1359/JBMR.0301262

[B20] Carpenter TO Imel EA Holm IA Jan de Beur SM Insogna KL A clinician's guide to X-linked hypophosphatemia J Bone Miner Res 2011 26 1381 1388 DOI: 10.1002/jbmr.340 10.1002/jbmr.340 21538511 PMC3157040

[B21] Akintoye SO Chebli C Booher S Feuillan P Kushner H Leroith D Cherman N Bianco P Wientroub S Robey PG Collins MT Characterization of gsp-mediated growth hormone excess in the context of McCune-Albright syndrome J Clin Endocrinol Metab 2002 87 5104 5112 10.1210/jc.2001-012022 12414879

[B22] Lee JS FitzGibbon E Butman JA Dufresne CR Kushner H Wientroub S Robey PG Collins MT Normal vision despite narrowing of the optic canal in fibrous dysplasia N Engl J Med 2002 347 1670 1676 10.1056/NEJMoa020742 12444181

[B23] Cutler CM Lee JS Butman JA FitzGibbon EJ Kelly MH Brillante BA Feuillan P Robey PG DuFresne CR Collins MT Long-term outcome of optic nerve encasement and optic nerve decompression in patients with fibrous dysplasia: risk factors for blindness and safety of observation Neurosurgery 2006 59 1011 1017 discussion 1017-1018 17143235 10.1227/01.NEU.0000254440.02736.E3

[B24] Misra M Cord J Prabhakaran R Miller KK Klibanski A Growth hormone suppression after an oral glucose load in children J Clin Endocrinol Metab 2007 92 4623 4629 10.1210/jc.2007-1244 17878248

[B25] Ruggieri P Sim FH Bond JR Unni KK Malignancies in fibrous dysplasia Cancer 1994 73 1411 1424 10.1002/1097-0142(19940301)73:5<1411::AID-CNCR2820730516>3.0.CO;2-T 8111708

[B26] Liu F Li W Yao Y Li G Yang Y Dou W Zhong D Wang L Zhu X Hu H Zhang J Wang R Chen G A case of McCune-Albright syndrome associated with pituitary GH adenoma: therapeutic process and autopsy J Pediatr Endocrinol Metab 2011 24 283 287 21823524 10.1515/jpem.2011.178

[B27] Geffner ME Nagel RA Dietrich RB Kaplan SA Treatment of acromegaly with a somatostatin analog in a patient with McCune-Albright syndrome J Pediatr 1987 111 740 743 10.1016/S0022-3476(87)80258-5 2889819

[B28] Christoforidis A Maniadaki I Stanhope R McCune-Albright syndrome: growth hormone and prolactin hypersecretion J Pediatr Endocrinol Metab 2006 19 Suppl 2 623 625 16789626 10.1515/jpem.2006.19.s2.623

[B29] Feuillan PP Jones J Ross JL Growth hormone hypersecretion in a girl with McCune-Albright syndrome: comparison with controls and response to a dose of long-acting somatostatin analog J Clin Endocrinol Metab 1995 80 1357 1360 10.1210/jc.80.4.1357 7714111

[B30] Galland F Kamenicky P Affres H Reznik Y Pontvert D Le Bouc Y Young J Chanson P McCune-Albright syndrome and acromegaly: effects of hypothalamopituitary radiotherapy and/or pegvisomant in somatostatin analog-resistant patients J Clin Endocrinol Metab 2006 91 4957 4961 DOI: 10.1210/jc.2006-0561 10.1210/jc.2006-0561 16984995

[B31] Akintoye SO Kelly MH Brillante B Cherman N Turner S Butman JA Robey PG Collins MT Pegvisomant for the treatment of gsp-mediated growth hormone excess in patients with McCune-Albright syndrome J Clin Endocrinol Metab 2006 91 2960 2966 DOI: 10.1210/jc.2005-2661 10.1210/jc.2005-2661 16720661

[B32] Brown RJ Kelly MH Collins MT Cushing syndrome in the McCune-Albright syndrome J Clin Endocrinol Metab 2010 95 1508 1515 10.1210/jc.2009-2321 20157193 PMC2853983

[B33] Carney JA Young WF Stratakis CA Primary bimorphic adrenocortical disease: cause of hypercortisolism in McCune-Albright syndrome Am J Surg Pathol 2011 35 1311 1326 DOI: 10.1097/PAS.0b013e31821ec4ce 10.1097/PAS.0b013e31821ec4ce 21836496 PMC4140081

[B34] Kirk JM Brain CE Carson DJ Hyde JC Grant DB Cushing's syndrome caused by nodular adrenal hyperplasia in children with McCune-Albright syndrome J Pediatr 1999 134 789 792 10.1016/S0022-3476(99)70302-1 10356155

[B35] Wood LD Parsons DW Jones S Lin J Sjoblom T Leary RJ Shen D Boca SM Barber T Ptak J Silliman N Szabo S Dezso Z Ustyanksky V Nikolskaya T Nikolsky Y Karchin R Wilson PA Kaminker JS Zhang Z Croshaw R Willis J Dawson D Shipitsin M Willson JK Sukumar S Polyak K Park BH Pethiyagoda CL Pant PV Ballinger DG Sparks AB Hartigan J Smith DR Suh E Papadopoulos N Buckhaults P Markowitz SD Parmigiani G Kinzler KW Velculescu VE Vogelstein B The genomic landscapes of human breast and colorectal cancers Science 2007 318 1108 1113 10.1126/science.1145720 17932254

[B36] Mauras N Blizzard RM The McCune-Albright syndrome Acta Endocrinol Suppl (Copenh) 1986 279 207 217 3465162 10.1530/acta.0.112s207

[B37] McGuffin WL Jr Sherman BM Roth F Gorden P Kahn CR Roberts WC Frommer PL Acromegaly and cardiovascular disorders. A prospective study Ann Intern Med 1974 81 11 18 4276173 10.7326/0003-4819-81-1-11

[B38] Bajpai A Greenway A Zacharin M Platelet dysfunction and increased bleeding tendency in McCune-Albright syndrome J Pediatr 2008 153 287 289 10.1016/j.jpeds.2008.02.045 18639732

[B39] Landis CA Masters SB Spada A Pace AM Bourne HR Vallar L GTPase inhibiting mutations activate the α chain of G_s_ and stimulate adenylyl cyclase in human pituitary tumours Nature 1989 340 692 696 10.1038/340692a0 2549426

[B40] Fang Z Mukai H Nomura K Shinomiya K Matsumoto S Kawaguchi N Kitagawa T Kanda H Establishment and characterization of a cell line from a malignant fibrous histiocytoma of bone developing in a patient with multiple fibrous dysplasia J Cancer Res Clin Oncol 2002 128 45 49 10.1007/s00432-001-0295-0 11862471 PMC12164401

[B41] Kyriakos M McDonald DJ Sundaram M Fibrous dysplasia with cartilaginous differentiation ("fibrocartilaginous dysplasia"): a review, with an illustrative case followed for 18 years Skeletal Radiol 2004 33 51 62 10.1007/s00256-003-0718-x 14647989

[B42] Ozaki T Lindner N Blasius S Dedifferentiated chondrosarcoma in Albright syndrome. A case report and review of the literature J Bone Joint Surg Am 1997 79 1545 1551 9378741 10.2106/00004623-199710000-00012

[B43] Yalniz E Er T Ozyilmaz F Fibrous dysplasia of the spine with sarcomatous transformation: a case report and review of the literature Eur Spine J 1995 4 372 374 10.1007/BF00300303 8983661

[B44] Beuerlein ME Schuller DE DeYoung BR Maxillary malignant mesenchymoma and massive fibrous dysplasia Arch Otolaryngol Head Neck Surg 1997 123 106 109 10.1001/archotol.1997.01900010116019 9006514

[B45] Hoshi M Matsumoto S Manabe J Tanizawa T Shigemitsu T Izawa N Takeuchi K Kawaguchi N Malignant change secondary to fibrous dysplasia Int J Clin Oncol 2006 11 229 235 10.1007/s10147-006-0559-4 16850130

[B46] Ruggieri P Sim FH Bond JR Unni KK Malignancies in fibrous dysplasia Cancer 1994 73 1411 1424 10.1002/1097-0142(19940301)73:5<1411::AID-CNCR2820730516>3.0.CO;2-T 8111708

[B47] Kanazawa I Yamauchi M Yano S Imanishi Y Kitazawa R Nariai Y Araki A Kobayashi K Inaba M Maruyama R Yamaguchi T Sugimoto T Osteosarcoma in a pregnant patient with McCune-Albright syndrome Bone 2009 45 603 608 DOI: 10.1016/j.bone.2009.05.018 10.1016/j.bone.2009.05.018 19481621

[B48] Blanco P Schaeverbeke T Baillet L Lequen L Bannwarth B Dehais J Chondrosarcoma in a patient with McCune-Albright syndrome. Report of a case Rev Rhum Engl Ed 1999 66 177 179 10327499

[B49] Chanson P Dib A Visot A Derome PJ McCune-Albright syndrome and acromegaly: clinical studies and responses to treatment in five cases Eur J Endocrinol 1994 131 229 234 10.1530/eje.0.1310229 7921205

[B50] Present D Bertoni F Enneking WF Osteosarcoma of the mandible arising in fibrous dysplasia. A case report Clin Orthop Relat Res 1986 238 244 3456857

[B51] Lima GA Gomes EM Nunes RC Vieira Neto L Sieiro AP Brabo EP Gadelha MR Osteosarcoma and acromegaly: a case report and review of the literature J Endocrinol Invest 2006 29 1006 1011 17259799 10.1007/BF03349215

[B52] Baris D Gridley G Ron E Weiderpass E Mellemkjaer L Ekbom A Olsen JH Baron JA Fraumeni JF Jr Acromegaly and cancer risk: a cohort study in Sweden and Denmark Cancer Causes Control 2002 13 395 400 10.1023/A:1015713732717 12146843

[B53] Collins MT Sarlis NJ Merino MJ Monroe J Crawford SE Krakoff JA Guthrie LC Bonat S Robey PG Shenker A Thyroid carcinoma in the McCune-Albright syndrome: contributory role of activating Gs alpha mutations J Clin Endocrinol Metab 2003 88 4413 4417 10.1210/jc.2002-021642 12970318

[B54] Tanabeu Y Nakahara S Mitsuyama S Ono M Toyoshima S Breast cancer in a patient with McCune-Albright syndrome Breast Cancer 1998 25 175 178 11091644 10.1007/BF02966691

[B55] Huston TL Simmons RM Ductal carcinoma in situ in a 27-year-old woman with McCune-Albright syndrome Breast J 2004 10 440 442 10.1111/j.1075-122X.2004.21490.x 15327499

[B56] Jenkins PJ Cancers associated with acromegaly Neuroendocrinology 2006 83 218 223 10.1159/000095531 17047386

[B57] Arik N Biriken D Akpolat T Sungur C Coskun C Basoglu T Keskin M Sahin M Toller MO Severe hyperparathyroidism associated with fibrous dysplasia: a case report Nephron 1996 74 481 482 10.1159/000189380 8893201

[B58] Braccini F Bacciu A Bruzzo M Pech-Gourg F Thomassin JM Craniofacial fibrous dysplasia associated with primary hyperparathyroidism Acta Biomed Ateneo Parmense 1999 70 5 11 11402810

[B59] Caudill R Saltzman D Gaum S Granite E Possible relationship of primary hyperparathyroidism and fibrous dysplasia: report of case J Oral Surg 1977 35 483 490 266063

[B60] Cavanah SF Dons RF McCune-Albright syndrome: how many endocrinopathies can one patient have? South Med J 1993 86 364 367 8451681

[B61] Ehrig U Wilson DR Fibrous dysplasia of bone and primary hyperparathyroidism Ann Intern Med 1972 77 234 238 4641656 10.7326/0003-4819-77-2-234

[B62] Hammami MM al-Zahrani A Butt A Vencer LJ Hussain SS Primary hyperparathyroidism-associated polyostotic fibrous dysplasia: absence of McCune-Albright syndrome mutations J Endocrinol Invest 1997 20 552 558 9413810 10.1007/BF03348018

[B63] Benedict PH Endocrine features in Albright's syndrome (fibrous dysplasia of bone) Metabolism 1962 11 30 45 13867139

[B64] Favilla C Abel T Kelly MP Chronic Galphas signaling in the striatum increases anxiety-related behaviors independent of developmental effects J Neurosci 2008 28 13952 13956 10.1523/JNEUROSCI.4986-08.2008 19091983 PMC2688724

[B65] Kelly MP Stein JM Vecsey CG Favilla C Yang X Bizily SF Esposito MF Wand G Kanes SJ Abel T Developmental etiology for neuroanatomical and cognitive deficits in mice overexpressing Galphas, a G-protein subunit genetically linked to schizophrenia Molecular psychiatry 2009 14 398 415 347 10.1038/mp.2008.124 19030002 PMC3312743

[B66] Kelly MP Cheung YF Favilla C Siegel SJ Kanes SJ Houslay MD Abel T Constitutive activation of the G-protein subunit Galphas within forebrain neurons causes PKA-dependent alterations in fear conditioning and cortical Arc mRNA expression Learning & Memory 2008 15 Cold Spring Harbor, NY 75 83 10.1101/lm.723708 PMC2216679 18230676

[B67] Bourtchouladze R Patterson SL Kelly MP Kreibich A Kandel ER Abel T Chronically increased Gsalpha signaling disrupts associative and spatial learning Learning & Memory 2006 13 Cold Spring Harbor, NY 745 752 10.1101/lm.354106 PMC1783628 17142304

[B68] Maxwell CR Liang Y Kelly MP Kanes SJ Abel T Siegel SJ Mice expressing constitutively active Gsalpha exhibit stimulus encoding deficits similar to those observed in schizophrenia patients Neuroscience 2006 141 1257 1264 10.1016/j.neuroscience.2006.04.028 16750890 PMC3311921

[B69] Kelly MP Isiegas C Cheung YF Tokarczyk J Yang X Esposito MF Rapoport DA Fabian SA Siegel SJ Wand G Houslay MD Kanes SJ Abel T Constitutive activation of Galphas within forebrain neurons causes deficits in sensorimotor gating because of PKA-dependent decreases in cAMP Neuropsychopharmacology 2007 32 577 588 10.1038/sj.npp.1301099 16738544 PMC3303872

[B70] Gould TJ Bizily SP Tokarczyk J Kelly MP Siegel SJ Kanes SJ Abel T Sensorimotor gating deficits in transgenic mice expressing a constitutively active form of Gs alpha Neuropsychopharmacology 2004 29 494 501 10.1038/sj.npp.1300309 14694347 PMC3348581

